# Biophilic Design as an Important Bridge for Sustainable Interaction between Humans and the Environment: Based on Practice in Chinese Healthcare Space

**DOI:** 10.1155/2022/8184534

**Published:** 2022-07-06

**Authors:** Yang Zhao, Qinchuan Zhan, Tiancheng Xu

**Affiliations:** ^1^College of Art and Design, Shaanxi University of Science & Technology, Xi'an, China; ^2^Key Laboratory of Acupuncture and Medicine Research of Ministry of Education, Nanjing University of Chinese Medicine, Nanjing, China

## Abstract

Since the COVID-19 epidemic, there has been an increased need for well-being and sustainable development, making biophilic design in hospital environments even more significant. However, after investigation, it was found that in many countries including China, the biophilic design of some hospitals is seriously absent, while other parts have the integration of biophilic design, but the standardization and recognition are not high. By restoring the interaction between buildings and nature, biophilic design improves the quality of environments and the health of users. The basic theoretical framework of environmental psychology is followed in this research. The health promotion mechanism, applicable natural features, and relative health advantages of hospital space and environment biophilic design are first investigated. Furthermore, according to the current status of biophilic design applications in the 12 hospitals that have the closest interaction between people and the environment. Combined with the professional and functional requirements of the healthcare spaces and the users' special demands, we propose appropriate update design methods. The goal of this study was to present ideas for healthy and efficient space environment design and to inspire sustainable environmental design for future healthcare environments.

## 1. Introduction

The normalization and recurrence of the COVID-19 have made the development of the healthcare spaces to become a global concern. In addition to balancing hygiene, efficiency, and equity, the design of healthcare spaces needs to be more integrated with the concept of sustainability, thus serving the health of all humanity in the context of the carbon neutral era [1]. As the number of hospital building projects grows, health administrators are progressively adopting green initiatives and ecologically friendly techniques. According to the federal Office of the Environment Executive (OFEE), “Sustainable hospitals can be defined as the practice of designing, constructing, operating, maintaining, and removing buildings in ways that conserve natural resources and reduce pollution [2].” The healthcare spaces have become one of the most visible venues for the green building movement as a result of concerns about hospitals' environmental implications. In the realm of architecture and design, there has been an increasing interest in the influence of nature on people in buildings [3, 4]. Biophilic design, which is strongly related to people's emotional experiences, is thought to play a significant role in this loop [5]. As an environmental design philosophy that promotes public health through the healing effects of nature, biophilic design interprets the relationship between nature, space environment, and human health from the perspectives of biology and psychology [6]. In the past decade, there has been a substantial increase in research related to biophilic design published by scholars from various countries [7], with applications ranging from interior design and architectural design to parks, streetscapes, schools, and urban design [8, 9]. Simultaneously, a group of researchers is looking into how the use of biophilic design in buildings and interior spaces improves patients' health and well-being [10]. Depression and mental health disorders are the main cause of disability globally, according to the World Health Organization, and because people spend 90% of their time in buildings [11], environmental design poses both difficulty and opportunity for designers [12].

In the context of COVID-19, people are forced to stay indoors in closed environments for extended periods, leading to heightened anxiety about the indoor environment and an urgent need to create medical spaces that promote emotional and physical well-being [13]. As a central place for the restoration of human health, hospitals are also working to create health-friendly spatial environments through the power of nature [14]. The main participants in medical activities, such as nurses and patients, are in a special state of intense concentration and are relatively fragile and sensitive [15]. The quality of the healthcare spaces' spatial environment is directly related to the effectiveness of medical treatment. And the natural attributes that biophilic design can give to the healthcare space's spatial environment and the health benefits it can create are highly compatible with the functions of the hospital and the demands of its users [16]. The existing literature suggested the various psychological and cognitive benefits of biophilic design, such as helped to have a positive impact on the patient's physiology and psychology [10], reduced negative emotions such as panic and anxiety [17], stimulated the patient's body's potential and promoting a return to health [16], reduced staff stress and fatigue [18], improved productivity, increased security in the environment [19], and negative associations with mortality [20].

To achieve restorative environmental design, real sustainability in healthcare environments should integrate low environmental impact design with biophilic design [21]. However, there is a lack of consistency in current research and practice in this area. Several issues with biophilic design have been observed in existing healthcare environments in China, according to the author's research. Natural resources are undervalued and in short supply. And some hospitals that use natural elements blindly copy them without appropriate selection and application according to the specific situation, lacking a systematic design theory to guide them, and ignoring the positive influence natural spaces bring to people. To seek the direction of sustainable design of healthcare spaces, as the starting point of creating a healthy environment for people, this research analyzes the application mode and existing problems of 12 healthcare spaces in China from the perspective of biophilic design and proposes suitable design solutions. Furthermore, given the size of the reaction to COVID-19 and its impact on health systems and the economy, constructive research action is crucial to reducing future health system restrictions and ensuring high-quality, accessible, and sustainable services [22].

The research is organized as follows: the introduction is presented in Section 1.  Section 2 analyzes the materials and methods of the proposed work.  Section 3 discusses the literature review. In Section 4, case study on biophilic design of healthcare spaces in China was explored in depth. In Section 5, the proposed methods are compared with previous concepts and made the results. Finally, in Section 6, the research work is concluded.

## 2. Materials and Methods

This paper conducted a literature analysis and a case study to establish the spatial design characteristics of healthcare settings using the biophilic design principle. The following is a detailed description of the research method and scope: we first outlined the positive effects of nature on human health, as well as the concept, elements, and patterns of biophilic design, based on the literature review. Second, to integrate and classify similar or flawed items in the biophilic design model in Chinese healthcare spaces from the case study results through field research and structured interviews to identify the characteristics and problems. Third, the transformed medical space environment was designed using software, including the 2021 edition of SketchUp, Lumion10 version, Enscape3.0 version, and AutoCAD2020. SketchUp is a 3D modeling software used in the interior design industry. Lumion is a real-time 3D visualization tool that covers areas including architecture, planning, and design. Enscape3.0 is used to render models. AutoCAD2020, or computer-aided design, uses computers and their graphics equipment to help designers with their design work. In the practical session, we used the concept of biophilic design to carry out an initial evaluation and update of a selected healthcare space environment.

## 3. Literature Review

### 3.1. Natural Elements and Human Health

Nature's therapeutic effect on physical and mental health has long been discussed. There are numerous direct and indirect relationships between human health and nature. Nature connectedness, in addition to meeting basic human requirements (such as food and natural resource availability), heals or mitigates the majority of ailments and can be considered a health resource (which keeps people healthy) [23]. The earliest site of physical healing by nature was the Sanctuary of Asklepios at Epidaurus, on a hill with fresh air and lush views was a rehabilitation centre in ancient Greece in classical times [24]. Professor Clair Cooper Marcus pointed out in her book *Healing Gardens: Therapeutic benefits and design recommendations*: “90% of garden users experience a positive change in their mood after taking a rest outside [25].” Ester M Sternberg wrote in her book *Healing Spaces: the science of place and well-being*: “When you see a scene that everyone likes, such as a beautiful view, sunset, woods, dormant nerve cells will become active.” Your mind is buzzing like a morphine addict. Both authors agreed that being in or observing a natural environment caused positive changes in the mind and body. As a source of healing and source of inspiration, nature plays an important role in the identity of people and the development of its sense of place.

The development of modern science and technology and the process of industrialization have brought about the transformation of human's view of nature to mechanistic, and the integrity and systematicness of nature have been broken in human cognition, which has led to essential changes in the relationship between human and nature. “The epidemic has made us understand that human health is inextricably linked to the health of natural ecosystems,” experts from the World Wide Fund for Nature (WWF) said. Human activities such as excessive and unnecessary production are primarily to blame for the calamities we have witnessed. We need to reverse this vicious cycle and protect and restore healthy ecosystems that thrive [26].

### 3.2. Biophilic Design

The international practice of biophilic design has a significant geographical dimension due to many factors such as regional development and socio-economic levels and culture. The 2006 conference in Rhode Island, USA, was the beginning of the formal introduction of biophilia into the field of environmental design, exploring the value and implementation strategies of its integration in cities and buildings [8]. In 2008, with the publication of *Biophilic Design: The Theory, Science, and Practice of Bringing Buildings to Life*, the term “biophilic design” was officially named and established. Biophilic design is about learning from nature and creating artificial environments that support and revive human biophilic nature by recreating, using, modeling, and extracting nature [27]. Kellert et al. proposed four basic principles for biophilic design in the same year: first, the importance of repeated and continuous engagement with nature; second, a focus on human adaptation to the natural world; third, encouraging emotional attachment to specific environments and places; fourth, promoting positive interaction between people and nature, encouraging an expanded sense of relationship and responsibility between human and natural communities; fifth, encouraging mutually reinforcing, interconnected and integrated architectural solutions [28]. William Browning proposed 14 biophilic design patterns in 2014 (Browning, Ryan, and Clancy 2014) in Table 1, and Stephen Kellett and Elizabeth Calabrese proposed 24 biophilic design strategies in 2015 [29] in Table 2. A comparative analysis of the two reveals that the design approach develops in three ways: the use of real natural elements, the abstraction and extraction of natural elements or characteristics, and the deduction and transformation of the relationship between man and nature. William Browning categorizes the first two components in terms of their homogeneity, reflecting a more simplified result, and adds to the third two features of “mystery” and “adventure.” By now, biophilic design has become a mainstream design approach in the field of architecture, widely accepted and used, and even included in the measurement criteria for evaluating the built environment. Biophilic design is not simply the transplantation of any natural elements into the spatial environment, but the translation of selected natural elements that have a positive effect on humans into concrete or abstract design language is based on the expansion of the connotation of natural elements, and their integration into each other and their application to the spatial environment in an effective way.

In conclusion, the biophilic design of buildings and urban environments abroad started early and has taken shape. To some extent, economically developed cities have launched biophilic design study and practice, demonstrating that biophilic design has become a significant trend in urban design. Although China's urban buildings are gradually adopting concepts like green ecology and sustainable development, many of the related designs still fall short of biophilic. This demonstrates that China is still systematically applying biophilic theories, models, and procedures. Many of the existing successful schemes are from Western countries. It is critical to learn from international advanced thoughts and experiences and investigate biophilic design methodologies that are appropriate for China's unique circumstances.

### 3.3. Biophilic Design in Healthcare Spaces

Ulrich conducted some of the earliest research into the application of biophilic design in healthcare spaces in the 1980s. His research found that patients in rooms overlooking green areas had shorter postoperative hospital stays and used less pain medication than patients in similar rooms but overlooking the built environment [31]. Following international investigations, it was shown that 95% of patients and families that were exposed to nature had lower stress levels, more positive attitudes, and improved coping skills [32]. Biederman and Vessel suggested that plants in healthcare spaces and roof gardens could reduce patients' pain, anxiety in therapeutic psychology in 2006 [33]. Eisen et al. conducted a study of art preferences among pediatric inpatients, which showed that children of different ages and genders did not differ much in their choice of artwork. They preferred nature art to abstract art, with nearly 75% preferring nature art (forests with lakes and deer) or impressionistic nature scenes (beaches with waves) [17].

Natural materials can improve patients' perceptions of their surroundings and their recovery from disease. This is because natural materials improve optical effects (by absorbing more light than they reflect) and have a favorable impact on olfactory comfort (through essential oils), creativity, overall health, and the immune system [34]. The famous German geographer and explorer Alexander von Humboldt emphasized the role of gardens in healing, suggesting that design should be integrated with nature, thus enhancing the quality of the existing environment [35]. The Sir Robert Ogden Macmillan Cancer Centre's chemotherapy space was created in the shape of a long cobblestone in response to patients' photosensitive and olfactory drug reactions, providing a long and soothing view of the patient's dizziness [36]. In 2020, Dushkova and Maria's biophilic-inspired “restorative healthcare environment design extends the focus from the outdoor landscape to the indoor architectural space.” Extensive experimental data showed that biophilic design in terms of natural light, greenery, green windows, outlook spaces, natural sounds, aromas, water features, real marine life, visual comfort, and a sense of personal control can all have a positive effect on promoting positive emotions and accelerating recovery [23]. Healthcare spaces are the core place for rehabilitation, and the study of the biophilic design of medical space environments has positive significance for improving the health of patients. The biophilic design research system of medical space is not yet perfect, and the norms and methods for the application of natural elements need to be further optimized in practice.

## 4. Case Study on Biophilic Design of Healthcare Spaces in China

Some hospitals in China were chosen for field research on the healthcare sector's spatial environment. The variety of hospital kinds was rather extensive, covering practically every aspect of medicine. Biophilic design applications were examined in some nodes with high foot traffic in hospital public spaces using field surveys and structured interviews. The aim was to identify the deficiencies and shortcomings of current healthcare institutions, propose corresponding solutions, and identify the characteristics of the biophilic design model for healthcare spaces.  Table 3 shows the basic information of 12 national hospitals with a floor area of 50,000 square meters or more.

Based on the selection of hospitals, the nodes with the high pedestrian flow in the hospital environment were selected, and seven nodes in the medical space, namely, the lobby reception, registration, and payment, waiting area, corridor, CT room, inpatient ward, and hospital lift, were extracted for study. The details of each node are shown in the following Table 4.

Through field research in a number of the above hospitals, it is possible to summarise the current biophilic design issues within the healthcare environment: the first is that the natural element is not valued and is severely lacking. Second, some hospitals that use natural elements blindly copy them without appropriate selection and application according to the specific situation, lacking a systematic design theory to guide them, and ignoring the positive influence natural spaces bring to people. Finally, the design approach's simplicity within some healthcare environments does not allow for efficient, rapid, and sustained action on human physiology. In terms of validity, the integration of individual natural elements into the hospital's spatial environment is isolated and stagnant, and the elements are not sufficiently appealing to people. In some healthcare environments where the principles of biophilic design are applied, it can be seen that such spaces not only relieve physical fatigue and reduce depression but also stimulate good moods and make patients cooperate with the examination and treatment.

The characteristics of biophilic design patterns in healthcare environments given in the literature and case studies were used to create 27 survey items in three patterns. To enhance respondents' comprehension, the questionnaire included photos of each scenario as well as the effect of applying the features. There were 240 responses, with a significant proportion of men (55%) and those aged 41 or older (41%) and patients (57.5%). The overall characteristics of survey respondents are shown in Table 5.

The importance of biophilic design patterns in healthcare spaces are shown in Table 6. The overall mean of the importance assessment was 4.08, which represents a consensus among the respondents on the importance of biophilic design. The importance of biophilic design patterns above the total mean was highest for “experience mode of natural sense of space” (4.24), followed by “direct nature experience mode” (4.09), and “indirect nature experience mode” (3.83).

As for the mean of importance by item, the highest was “create a relatively inward-looking space environment that resembles a cave in nature, with a long-distance open view in the foreground, and a sense of wrapping from overhead, behind, and on both sides, such as entrances with overhangs and colonnades balconies, sofa seats, etc.” (4.58), followed by “real plants or specimens, green roofs, green walls” (4.55), and “the natural building or decorative materials such as wood, stone, wool, cotton and leather, bamboo, rattan” (4.47). And each element of the biophilic design pattern brings different physiological, psychological, and perceived benefits to the user. From the results of the public's assessment of the importance of the elements of biophilic design patterns, it can be concluded that they need small spaces that can provide shelter and lookout and prefer direct natural elements. When planning the design of the medical space in the future, the biophilic design pattern characteristics of the healthcare spaces that are relatively important in the analysis results should be considered.

## 5. Results

This chapter will present appropriate design strategies for the problems found in the biophilic design of healthcare spaces in the research.

### 5.1. The Overall Design Idea of Transformation

**Figure 1:**
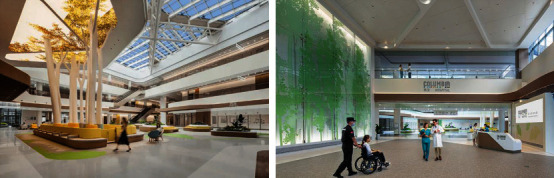
Kaiyi Hospital's Hall biophilic design update renderings.

The outpatient hall and other locations guidance systems of some hospitals such as Jiangsu Provincial Hospital are relatively single, the overall color is single, the use of natural elements is less and stiff, and the introduction of natural light is limited. In response to the above problems, further skylights can be added in the outpatient halls and corridors to introduce natural light into the indoor space, which can not only relieve the pressure of patients but also save electricity and reflect the concept of sustainable development. Second, the use of floor-to-ceiling windows in the waiting room should be raised in an appropriate amount to increase the gentle combination of indoor and outdoor spaces, so that patients waiting can enjoy the view of the outdoors from the comfort of their own home (especially by making good use of the landscape resources of the adjacent mountains). Third, indirect natural elements are incorporated into the stylistic design by reducing the use of arrow-like directional signs and using green, blue, yellow, or faceted signs to guide and divide spaces. In the Kaiyi Hospital's hall design, designers have shaped the hospital hall into a well-lit and ventilated atrium. The lounge area in the hall is arranged to look like a living room, with sunlight illuminating the whole hall through the skylight on the roof, and with indoor greenery, trying to create a homely atmosphere and a warm resting space for doctors and patients. In the lobby, the supporting beams are used as a base combined with the top lighting to create a decorative design in the shape of a ginkgo tree, which symbolizes strength, hope, and resilience. At the same time, its color reflects the representative color of Jiaxing, namely, the Ginkgo Avenue on Swan Lake Road in autumn, incorporating local cultural elements into a modern large general hospital. These qualities were distilled into an abstract design language by the designers and used in the hospital interior design (Figure 1).

### 5.2. The Partial Transformation of the Consultation Room

**Figure 2:**
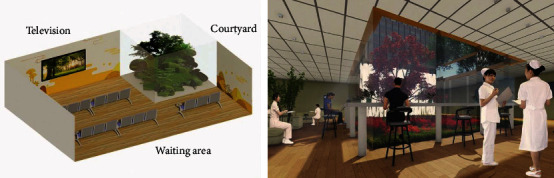
Rendering of the updated biological design of the waiting area.

The overall spaces of the waiting area in some hospitals are relatively closed, mostly in a room with glass on one side, which lacks communication with the outdoor environment and has a relatively depressing atmosphere, which is unfavorable for both doctors and patients. To address the aforementioned difficulties, a small patio-style garden can be added to the waiting area, introducing natural light through the patio and floor-to-ceiling windows so that patients can enjoy the external beauty even when they are inside. The two-story cedar shingle walls and windows of various offices look out over the space, which is filled with plants, the majority of which are medicinal species. Second, the paths leading to the two seats at either end are unusual water features made of concrete stepping stones set in moss [2]. Multimedia equipment is added to the wall facing the waiting patients, decorated with geometric patterns of the golden ratio. Finally, add several cave-like lounge sofas and play soft music containing natural elements such as the sound of water and birdsong, which can help to alleviate the fatigue of people and relieve the patient's anxiety (Figure 2).

### 5.3. Renovation of the Functional Examination Unit

The CT room is undecorated, and the white walls and cold machines can put patients in a depressing and frightening mood, especially as the examinations often allow only the patient to be alone, while examinations such as fMRI take 30 minutes or more. To address these concerns, the walls can be painted in a natural-elements design, and vegetation can be put to the room to help the patient relax. Since the patient is in a lying position during the examination, the eyes are focused on the top, natural patterns such as the sky or woods can be added to the ceiling, while natural sounds such as fountains, streams, waves, birdsong, and rain can be added to soothe the patient through a combination of audio and visual stimulation [37].

### 5.4. Ward Transformation

**Figure 3:**
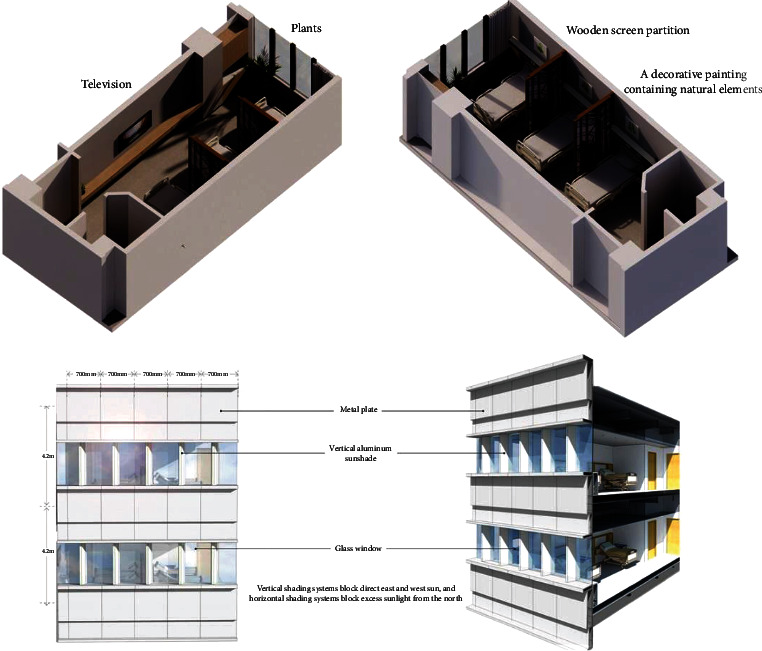
Ward biophilic design update renderings.

The overall color palette of the ward space is predominantly white, lacking color variation and plants, while the furniture and cabinets are mostly made of metal and plastic, lacking a sense of intimacy. The beds in the multiperson ward are separated from each other only by bed curtains and lack personal space. To address these concerns, natural elements can be used to beautify the wards by including appropriate plants and animals, such as green buckets and goldfish. Create a unique natural element in the landscape by forming the walls in the shape of a natural environment, such as a beehive, and then adding suitable plants to the individual units. Second, instead of bed curtains, screens with pulleys can be used. Screens of rigid material are more capable of dividing space than fabric bedroom curtains, and the pulleys ensure that the flow of space is unobstructed [28]. The ward unit adopts a zigzag plane, and green platforms are arranged in the recesses in the plane so that there is a natural landscape outside each window. Combined with the external multilevel healing garden, patients on each floor have the opportunity to have direct contact with nature and use the “direct experience mode” with nature in the biophilic design to enhance the healing properties of the interior space. In addition, through the overall consideration of the window size and the location of the hospital bed, the window ventilation only serves the nearby patients, reducing the risk of mutual infection. The windows also feature removable louvers to maximize daylight and minimize glare, shielding patients from low-angle sunlight in the morning and evening. And a forward-facing eave can be added to the window to give the patient a sense of perspective (Figure 3).

### 5.5. Garden Renovation

**Figure 4:**
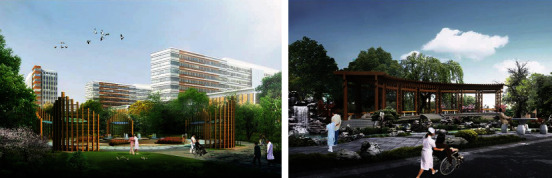
Healing garden biophilic design update renderings.

The survey found that some Chinese hospitals did not realize the role of healing gardens, several healing garden space is less functional, the configuration of plants and so on is relatively single, and the landscape modes are not sparse and dense. First, a multifaceted composite landscape system can be introduced into the garden, such as different scales and types of nonirritating odor plants, to create spatial places with different experiential sensations and to increase complexity and connectivity. The use of plants and geometric cuts and microtopographical enclosures creates a private and secluded place that adds a sense of mystery and provides a sense of refuge for patients [38]. Tree-shrouded paths lead people's eyes to distant views and landmarks, giving depth and coherence to the space of this journey. This atmosphere allows people to control their emotions and restore their focus by providing a momentary getaway, a sense of “distance.” Then there is the introduction of water features, which are classified as either dynamic or static depending on their qualities. Dynamic water features such as cascading water and streams can create a natural and dynamic water ecology and bring vitality to patients. Landscapes such as water mists and fogs can increase the humidity of the air in the hospital environment and create a microclimate of comfortable spaces. Patients can interact with the water features to create a pleasant emotional experience. Third, the introduction of animal therapy, the inclusion of animal elements, can promote interaction between people and nature and increase the emotional connection of the place. The interaction and play of patients with animals can increase patients' sense of belonging to the hospital space, which in turn relieves patients' physical exhaustion and pain (Figure 4).

## 6. Conclusions and Discussion

To achieve true sustainability in healthcare facilities, low environmental impact design should be combined with biophilic design (or positive environmental impact design), resulting in what is known as a restorative environmental design [28]. Biophilic design is based on the biophilic nature of human beings. It is a space-environment design concept that can boost human health, cognition, and productivity. The biophilic design idea highlights the relationship between nature, space environment, and human health from a biological aspect and can drive the long-term development of the healthcare spaces environment. To ensure safety and standardization, natural elements suitable for the healthcare spaces environment should be picked, and then prudent and effective design approaches should be used, all while not interfering with medical activities and completely addressing the demands of patients. The idea is to continuously stimulate the human body using a variety of sensory inputs like vision, hearing, touch, and smell. People's biophilia is stimulated, and doctors' and patients' health is enhanced, by leading the human body to create a response to natural components. This is a paradigm aimed at reestablishing a harmonious balance between humans and nature in the building environment. Biophilic design, within this concept, may represent the missing piece in sustainable design, which is still related to an understanding of nature as an ethical value rather than a physiologically determined condition.

To ensure the correct biophilic design of the healthcare space environment, to ensure the safety of patients' lives, and to avoid additional physical and psychological harm to patients during the treatment process, the biophilic design process must carefully select natural elements and use highly controlled design methods to avoid the possible increased risk of infection and physiological burden of natural elements. Based on the existing theoretical framework of biophilic design, this study analyzes the existing problems, health promotion mechanism, and specific associated health benefits of biophilic design through field investigations in 12 healthcare spaces in China. Then combined with the professional requirements of hospital functions and the special needs of users, the following three points of biophilic design are proposed:

### 6.1. Optimize the Interaction Mode between Direct or Indirect Natural Elements and People

In traditional medical spaces, natural elements such as greenery and animals are used for aesthetic and even Feng Shui reasons. In most of the hospitals and clinics we studied, greenery was used only as a landscape, at best for the short-term value of “removing formaldehyde” [39], but not for its value in optimizing the flow of care or even for its medical value [40]. Natural elements such as plants and natural light can themselves create order and hierarchy. The placement of plants in our design corresponds to, and even merges with, the flow design of medical appointments, serving as a good segregation and guide. And this is just the beginning of its job. The plants themselves can become the focus of attention for patients and doctors, especially in information-heavy waiting and registration areas, and the interplay of information screens and varied signage with the plant arrangement can serve to reduce anxiety among patients [41]. The introduction of animals is a bold design, but we were fortunate to see more than one site in the hospitals we researched that had adopted this element, such as the ornamental fish pond provided in the outpatient halls of the Jiangsu Provincial Hospital of Traditional Chinese Medicine. This design enhances the cognitive and reactive abilities of patients through the interaction of animals with them. Especially for dentistry, a department that causes more direct pain, the active character of the fish has a relaxing effect on patients [42]. Animals are especially important in the medical value of human interaction because their natural curiosity helps to provide additional healing space for parents with children, whose increased attention to animals reduces their attention to other things around them, such as crying children and anxious patients.

In addition, the use of indirect natural elements such as natural colors (e.g., soft colors such as green, blue, and yellow), natural materials (e.g., wood, stone, and bamboo), simulated light and air, natural geometry (fractals, golden ratio, golden spiral, etc.), natural associations (abstraction and symbolization of natural forms), and natural images (multimedia natural landscapes) in interior spaces also need to be based on the needs of the patient's healthcare experience. And to systematically examine the volume of consultations and the geographical distribution of specialties in the renovated premises, to improve the health of the staff in three directions: mental cognition, psycho-emotional, and physiological functions.

### 6.2. Increase the Emotional Connection of the Place and Provide an Interactive Place for Shelter and Lookout

Following extensive fieldwork, the research identified a lack of natural spatial experience models in healthcare settings. Hospitals are stressful places where both staff and patients want some private sanctuary to relieve their emotions. The advantage of the natural space experience model is that it is easier to create an immersive experience of nature by creating a spatial organization in an artificial environment that resembles that which exists in nature. Relevant natural elements include a sense of shelter and watchfulness, order and complexity, mystery, and contrast. In the actual design process, real natural objects, natural analogs, and products are often used to assist in creating a sense of natural space. This model not only helps to alleviate mental fatigue caused by continuous and intense work but it also promotes refocusing and intelligent recovery; it also helps to increase health care staff motivation, willingness to communicate with patients, and overall work effectiveness, all of which have a positive impact on patient recovery.

For example, healing gardens can provide a temporary sanctuary with a degree of privacy, offering a place to escape for patients who have been in a group living space. Create a relatively inward-looking space environment that resembles a cave in nature, with a long-distance open view in the foreground, and a sense of wrapping from overhead, behind, and on both sides, such as entrances with overhangs and colonnades balconies, sofa seats, etc., and it can increase patient's emotional attachment. Emotional attachment is a person's high identification with the environment, which helps patients to develop a series of positive emotions, such as relaxation, willingness to integrate into the treatment environment, and more confidence in the treatment plan.

### 6.3. Realize Systematic Compounding with Real and Diverse Designs

In terms of achieving effectiveness in the biophilic design of healthcare spaces, research has shown that real natural objects can have a more positive effect than simulated natural analogs, and that overly distorted natural analogs can cause boredom and resentment [28]. The combined health advantages of using several natural components in a systematic way are more important and conducive to the production of a sense of natural space and environment than the benefits of using individual natural elements alone [21]. The multisensory complex stimulation of the human body by natural elements is more attractive than the stimulation of a single sense. Elements of nature that attract active participation and physical activity are more influential than those that only allow for static appreciation of dwelling [43].

Considering the complexity of the spatial environment of the hospital and the specificity of the personnel, to enhance the effectiveness of the design: first, it should try to objectively and realistically display the diversified natural elements more understandably, moderately highlight the dynamic changes and attractiveness of the natural elements, and thus enhance the participation of the personnel; second, the biophilic design of each spatial interface is compounded. Patients are often supine when receiving treatment and resting, so the design should focus on the roof interface, which is often overlooked but has a high frequency of patient sighting; third, when it is difficult to make a direct connection between the imaging centre and the real exterior natural world due to the spatial limits imposed by the functional requirements of healthcare, reference can be made to the indirect natural experience model by systematically using natural materials such as logs to awaken tactile perception, electronic media, and virtual reality technology to reshape the audiovisual experience, and natural scents to activate the olfactory memory, creating a comprehensive fake natural experience.

Millions of people's health had improved significantly before the COVID-19 epidemic. However, additional work is needed to completely eradicate a variety of diseases and treat a variety of persistent new health challenges. Biophilic design is one of the efforts that need to be implemented, a concept that is difficult to implement in many developing countries and regions due to economic factors and research limitations. In line with the general trend of human evolutionary progress, the biophilic design is founded on genuine life impulses and survival laws. Biophilic design can contribute to the health promotion of hospital spaces and is in line with the requirements of sustainable development. With the development of mankind's understanding of the relationship between humans and nature, the natural elements with health-promoting potential can be further expanded in the future through more extensive and scientific empirical research, and their health-promoting effects on specific people in specific hospital spatial environments can be explored in a targeted manner, providing a more adequate theoretical basis and empirical guidance for the future biophilic design of hospital spatial environments.

## Figures and Tables

**Table 1:** The biophilic design methods from William Browning in 2014 [30].

Classification of design methods	Design elements
Apply natural elements	(1) Visual connections; (2) nonvisual connections; (3) irregular sensory stimuli; (4) heat and air currents; (5) water; (6) dynamic diffuse light; (7) natural systems
Simulation of natural analogs	(8) natural forms; (9) natural materials; (10) complexity and order
Construct the relationship between human and nature	(11) Prospect; (12) refuge; (13) mystery; (14) adventure

**Table 2:** The biophilic design methods from Stephen Kellert in 2015 [29].

Classification of design methods	Design elements
Direct nature experience	(1) light; (2) air; (3) water; (4) plants; (5) animals; (6) weather; (7) natural systems; (8) fire
Indirect nature experience	(9) natural patterns; (10) natural materials; (11) natural colors; (12) simulated natural light and natural ventilation; (13) natural shapes or forms; (14) natural associations; (15) information richness; (16) change of time; (17) natural geometry; (18) bionics
The experience of space and place	(19) foresight-shelter; (20) organize complexity; (21) integration; (22) transitional spaces; (23) mobility and wayfinding; (24) emotional connection of place

**Table 3:** General information of healthcare spaces in China was studied in the case.

The name of the hospital institution	Geographical location	Total area (m^2^)	Nature
Nanjing University of Chinese Medicine	Nanjing, China	55000	National General Hospital
Jiangsu Dental Hospital	Suzhou, China	63000	National Specialist Hospital
Jiangsu Province Hospital	Nanjing, China	20000	National General Hospital
Jiangsu Provincial Hospital of Medicine	Nanjing, China	49000	National General Hospital
Nanjing Children's Hospital	Nanjing, China	112500	National General Children's Hospital
Nanjing Maternity and Child Healthcare Hospital	Nanjing, China	20200	National General Hospital
Nanjing Brain Hospital	Nanjing, China	66000	National Specialist Hospital
Nanjing Medical University Affiliated Eye Hospital	Nanjing, China	20000	National Specialist Hospital
Jiangsu Province Reproductive Centre	Nanjing, China	33110	National Specialist Hospital
Xi'an Children's Hospital	Xi'an, China	75000	National General Hospital
Jinwan Hospital	Zhuhai, China	62000	National General Hospital
Zhongshan Hospital	Shanghai, China	96000	National General Hospital

**Table 4:** Characteristics of biophilic design elements and patterns in healthcare spaces of China.

	Research status		
Situation picture	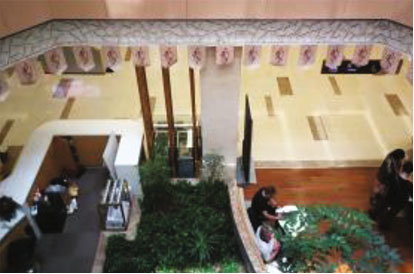	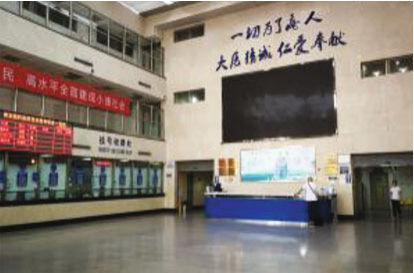	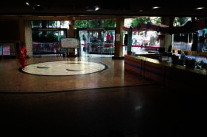
Name	Nanjing University of Chinese Medicine	Nanjing Brain Hospital	Nanjing Children's Hospital
Node	Lobby reception		
Behavior	Enter the lobby to look for the department, waiting		
Mood	Peaceful	Depression	Boredom
Biophilic design applications	Accelerate smooth recovery of physiological features using real plant elements in the direct nature experience model.	Lacking	Large glazed windows extend the view of the natural landscape outside the house and also help to increase the light inside the house.
Problem	Lack of organic interaction between clusters.	Lack of interactive design in the lobby and lack of natural elements.	Lack of a clear signage system to avoid confusion and congestion.
Situation picture	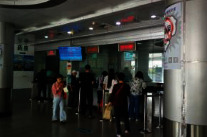	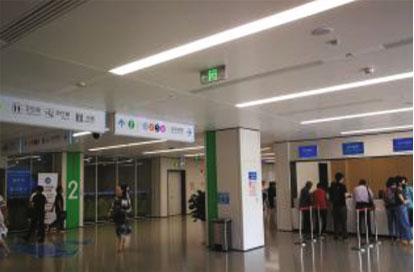	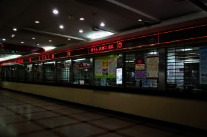
Name	Jiangsu Dental Hospital	Jiangsu Province Hospital	Nanjing Children's Hospital
Node	Registration and payment		
Behavior	Waiting inline		
Mood	Dull	Boredom	Tension
Biophilic design applications	Lacking	The use of natural colors such as green and wood gives a fresh, vibrant feel, and effectively divides the space.	Lacking
Problem	The overall color is metallic, cold, and monochrome.	Insufficient shade and proper privacy space.	Lack of natural elements.
Situation picture	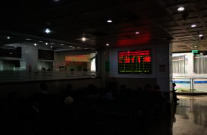	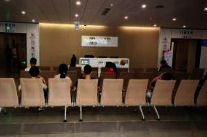	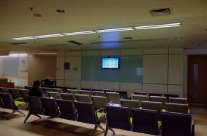
Name	Jiangsu Dental Hospital	Jiangsu Province Hospital	Jiangsu Province Reproductive Centre
Node	Waiting area		
Behavior	Waiting, consulting		
Mood	Depression	Peaceful	Somber
Biophilic design applications	Lacking	Both the walls and the seats are in carpenter's colors can be used to recreate the feeling of the forest and the plants.	Skylight for increased natural light.
Problem	The waiting area has no windows, and the lighting is weak, creating an overall dim atmosphere.	Lack of interactive elements.	The decorative dividing lines on the walls are crossed series a straight line, giving a serious and discreet impression.
Situation picture	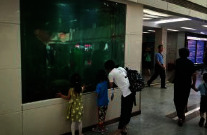	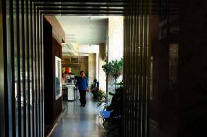	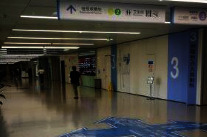
Name	Jiangsu Province Hospital	Jiangsu Province Reproductive Centre	Jiangsu Dental Hospital
Node	Corridor		
Behavior	Walking, searching, resting		
Mood	Pleasant	Comfortable	Boring
Biophilic design applications	A large aquarium with a variety of aquatic animals, where the creatures swim to add life and vitality.	The use of bamboo and greenery to create shade, large windows to increase natural light.	A considerable amount of sky blue has been used to help create an overall bright and vibrant interior.
Problem	The single form of interaction.	Lack of multisensory indirect natural elements.	Lack of multisensory indirect natural elements.
Situation picture	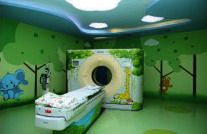	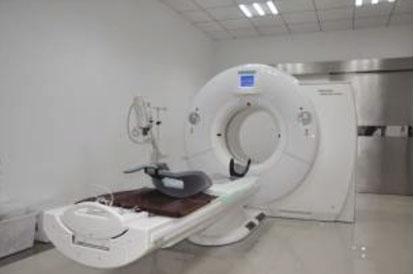	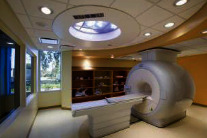
Name	Xi'an Children's Hospital	Jiangsu Province Hospital	Jinwan Hospital
Node	CT room		
Behavior	Body checking		
Mood	Funny	Tension	Comfortable
Biophilic design applications	The cartoonish design of the forest elements on the base and around the treatment equipment and the house makes it more accessible and enjoyable for children.	Lacking	Natural light, warm walls, outdoor greenery, and landscape paintings create a warm and welcoming atmosphere.
Problem	Raw use of natural elements.	Lack of biophilic design.	The lighting setup is a little complicated.
Situation picture	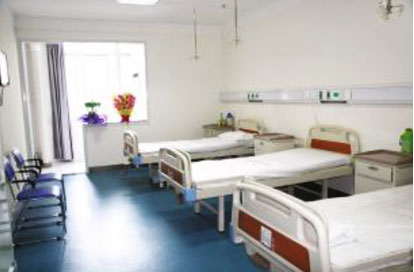	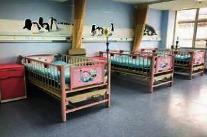	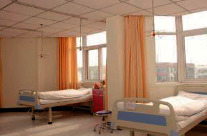
Name	Nanjing Medical University Affiliated Eye Hospital	Xi'an Children's Hospital	Nanjing Maternity and Child Healthcare Hospital
Node	Inpatient Ward		
Behavior	Hospitalization, visiting patients		
Mood	Torment	Lively	Comfortable
Biophilic design applications	Lacking	Natural elements such as animals and ocean glaciers set the mood, tension-relieving colors such as pink and blue.	The use of natural, warm colors creates a warm and inviting atmosphere.
Problem	The large areas of cold white and the hard iron chairs on the walls tend to bring depression to people recovering from hospitalization.	Lack of multisensory direct and indirect natural elements.	Lack of multisensory direct and indirect natural elements.
Situation picture	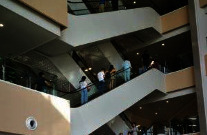	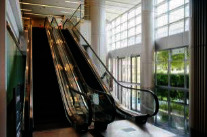	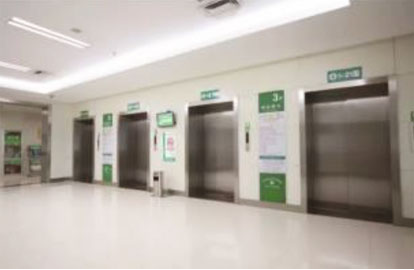
Name	Xi'an Children's Hospital	Zhongshan Hospital	Nanjing Brain Hospital
Node	Hospital lift		
Behavior	Walking, looking		
Mood	Boring	Peaceful	Boring
Biophilic design applications	Lacking	The large floor-to-ceiling windows not far from the lift bring in a lot of natural light into the medical space and help communication between the inner and outer spaces.	Lacking
Problem	Crowded surroundings monochromatic colors single layout.	Lack of multisensory direct and indirect natural elements.	Cold lifts and empty grounds give a dead feeling and are not conducive to healing.

**Table 5:** General characteristics of survey respondents.

Category	Item	*N*	%
Gender	Male	132	55
	Female	108	45
	Total	240	100

Age	Age 25-30	73	30
	Age 31-40	69	29
	Age 41 or older	98	41
	Total	240	100

Identity	Patient	137	57.5
	Hospital staff	58	24.2
	Others	45	18.3
	Total	240	100

**Table 6:** Importance of biophilic design patterns in healthcare spaces.

Biophilic design pattern	Associated natural elements	Biophilic design pattern characteristics	Impact on users			Mean of importance
			(a)	(b)	(c)	
Direct nature experience mode	Plant	Real plants or specimens, green roofs, green walls.	●	●	●	4.55
	Animal	Dedicated, safe, and effective space for animal-assisted therapy.	●	●	●	3.94
		The hospital waiting for the hall, restaurant, and other spaces are equipped with aquariums with a variety of aquatic animals.	●	●	●	4.13
	Natural light	Introduce natural light indoors by setting up roof gardens, sunken gardens, corridors with windows and skylights, offices, lounges, wards, etc.	●			4.26
	Water	Water bodies, fountains, constructed wetlands, small waterfalls, water sound.	●	●	●	4.11
	Air	Natural ventilation.	●	●		4.43
	Weather	Through the interface design of transition spaces such as building doors and windows, skylights, balconies, corridors, etc., people can know the weather conditions.	●	●	●	4.25
	Fire	A fireplace or a fire that simulates the light's color, motion, and temperature.	●			3.98
	Natural systems	The holistic nature is composed of plants, animals, water bodies, soil, rocks, etc., including topography, vegetation landscapes, and ecosystems.		●		4.23
Indirect nature experience mode	Natural images	Natural images are represented by photographs, paintings, sculptures, murals, videos, computer simulations, and other means.	●	●		4.11
	Natural sound	Play soft natural sounds of fountains, streams, waves, waterfalls, rain, wind, birdsong, and more.	●	●	●	3.43
	Natural materials	The natural building or decorative materials such as wood, stone, wool, cotton and leather, bamboo, and rattan.		●	●	4.47
	Natural color	Natural pastel shades of soil, rocks, plants, etc., avoiding strongly artificial, contrasting, and vibrating colors.		●	●	4.45
	Simulate light and air	Simulation of natural spectral and dynamic properties and natural ventilation.	●	●	●	4.13
	Natural form	Extraction of natural forms, such as plant patterns such as flowers and trees; animal forms such as shells and honeycombs.	●	●	●	3.54
	Natural association	Abstraction and symbolization of natural forms.		●		3.65
	Informative	Rich environmental information can activate the perception of the environment by various senses, such as vision, touch, and smell.	●	●	●	3.43
	Change of time	Visualization and characterization of changes over time, such as aging of materials and oxidation of metals.		●		4.38
	Natural geometry	The use of natural geometry, such as fractals, golden ratio, golden spiral, Fibonacci sequence, and dynamic symmetry.	●	●	●	3.54
	Integration	Numerous unique elements come together into a unified whole, including continuous spatial relationships, clear boundaries, functional, or formal focal points.		●		3.43
	Bionics	Imitation of nature to optimize performance, not simple morphological replication.		●		3.41
Experience mode of natural sense of space	The prospect-refuge	Create a relatively inward-looking space environment that resembles a cave in nature, with a long-distance open view in the foreground, and a sense of wrapping from overhead, behind, and on both sides, such as entrances with overhangs and colonnades balconies and sofa seats.	●	●	●	4.58
	Mobile wayfinding	Clear paths and exits and guidance systems ensure autonomous movement of the individual.		●		4.36
	Transition space	Clear boundaries and connectors to ensure an individual's sense of domain and control.		●		3.80
	Place emotional connection	Establish a connection between the individual and the local geography, history, culture, or ecological environment to enhance a sense of belonging and identity.	●	●	●	4.41
	Mystery	On the premise of ensuring safety and control, set up winding paths, or use plants to partially cover the building, blur some sensory information, and stimulate people's curiosity for further exploration.		●	●	3.94
	Order and complexity	Introduce a landscape system with multiple elements; adopt a hierarchical spatial structure; moderately use fractal patterns in nature; repeat motifs.	●	●	●	3.65

(a) represents physiological benefits, (b) representing psychological benefits, and (c) representing perceived benefits.

## Data Availability

The datasets used during the current study are available from the corresponding author on reasonable request.
